# Possible Facilitative Effects of Repeated Anodal Transcranial Direct Current Stimulation on Functional Outcome 1 Month Later in Schizophrenia: An Open Trial

**DOI:** 10.3389/fpsyt.2017.00184

**Published:** 2017-09-29

**Authors:** Zui Narita, Takuma Inagawa, Kazuki Sueyoshi, Crystal Lin, Tomiki Sumiyoshi

**Affiliations:** ^1^Department of Psychiatry, National Center Hospital, National Center of Neurology and Psychiatry, Tokyo, Japan; ^2^Department of Clinical Epidemiology, Translational Medical Center, National Center of Neurology and Psychiatry, Tokyo, Japan

**Keywords:** brain stimulation, cognition, daily living skills, tDCS, functional outcome

## Abstract

Recent research on neuromodulation techniques, such as transcranial direct current stimulation (tDCS), for the treatment of schizophrenia has mainly focused on psychotic symptoms. We aimed to determine whether repetitive tDCS is efficacious in improving determinants of outcome, such as cognitive function, daily living skills, and depressive mood in patients with schizophrenia. Twenty-eight patients underwent tDCS (2 mA × 20 min) two times per day for 5 consecutive days. The anodal electrode was placed over the left dorsolateral prefrontal cortex while the cathodal electrode was placed over the right supraorbital region. One month after the last stimulation, there was a significant improvement on cognitive function, measured by the brief assessment of cognition in schizophrenia (*d* = 0.49). Significant effects were also shown on daily living skills (functional capacity), measured by the UCSD performance-based skills assessment-brief (*d* = 0.70). Depressive symptoms, measured by the Calgary depression rating scale, as well as psychotic symptoms measured by on the positive and negative syndrome scale positive and general psychopathology subscales also responded to the treatment (*d* = 0.38, *d* = 0.48, and *d* = 0.50, respectively). This is the first study to suggest that tDCS with the anodal electrode on the left prefrontal cortex improves functional capacity and depressive symptoms in patients with schizophrenia. These results may add to the concept that tDCS provides a strategy to enhance functional outcomes in patients with schizophrenia.

**Trial Registration:**

https://upload.umin.ac.jp/cgi-open-bin/ctr/ctr_view.cgi?recptno=R000018556, UMIN000015953.

## Introduction

Schizophrenia patients elicit psychotic symptoms, mood symptoms, and cognitive impairment (1–3). Specifically, cognitive function, such as learning memory, working memory, executive function, verbal fluency, and attention/information processing, are impaired in patients with the illness (4, 5). Functional capacity is defined as the potential to perform everyday living activities, which require financial competence, communication skills, and so on (6). By contrast, real-world functional outcomes (social function) are greatly affected by several factors, such as opportunities and incentives that influence functioning in everyday situations (7). These levels of functional outcomes (cognitive function, functional capacity, social function) have been reported to be associated with each other (8, 9).

In patients with schizophrenia, functional connectivity of the frontoparietal control network and inter-hemispheric connectivity are decreased, which may play an important role in the pathophysiology of impairment of higher order cognitive task-related activities (10–12). The dorsolateral prefrontal cortex (DLPFC) is reported to be related to these circuits, and shows functional changes of cognitive function in schizophrenia (13, 14). Transcranial direct current stimulation (tDCS) is a feasible and safe method, using weak and direct electrical current to the brain through electrodes (15, 16). tDCS changes cortical excitability, modulated by glutamatergic activity *via* actions on catecholamine, acetylcholine and serotonin receptors (17–19). With this mechanism, tDCS over the left DLPFC has been suggested to modulate corticosubcortical/corticocortical pathways (20, 21).

The beneficial effect of tDCS on cognitive function has been reported. For example, Vercammen et al. observed that a subset of patients with schizophrenia, with greater variance in the active relative to the sham conditions, may respond to tDCS over the DLPFC (22). In addition, Schretlen et al. reported facilitative effects of tDCS over the left DLPFC on measures of working memory and aspects of verbal fluency relevant to word retrieval (18). Moreover, Hoy et al. found that repeated tDCS over the left DLPFC may enhance working memory in schizophrenia by restoring normal gamma oscillatory function (23). Furthermore, a sham-controlled randomized study demonstrated that repeated tDCS over the left DLPFC improved performance on the MATRICS Consensus Cognitive Battery (24). Thus, the DLPFC has been a target for studies investigating tDCS on cognitive function in schizophrenia (18, 22–24).

Although these studies indicate the facilitative effect of tDCS over the DLPFC on some domains of cognitive function, there is little information on whether tDCS would improve a higher level of functional outcome, e.g., daily living skill linked to cognitive function (functional capacity), in schizophrenia. We hypothesized that tDCS may also be effective in improving functional capacity in schizophrenia, since this level of functional outcome is associated with cognitive function, as mentioned above (8, 9). To our knowledge, no study has been attempted to determine whether tDCS directly improves functional capacity, or the improvement on other symptoms, such as psychosis, depression, would indirectly improves it. Based on these considerations, the primary aim of present study was to evaluate the effect of anodal tDCS over the left DLPFC on functional capacity in schizophrenia. Also, we sought to determine whether or not a putative improvement of functional capacity by tDCS would be related to changes of other clinical factors, such as cognition, psychotic symptoms, and depressive symptoms.

## Materials and Methods

### Participants

Inpatients or outpatients treated at National Center Hospital, National Center of Neurology and Psychiatry, were enrolled. Participants were recruited by psychiatrists’ referrals. They provided written informed consent before starting the trial.

Subjects met the following inclusion criteria:

(1) Meeting DSM-5 criteria for schizophrenia.
(2) Being 20 through 60 years old.
(3) Being able to sign and give consent.

Patients with any of the following diagnoses in accordance with clinical interview by psychiatrists were excluded from the study:

(1) Alcohol or substance disorder
(2) Traumatic brain injury
(3) Epilepsy

Twenty-eight subjects were enrolled, and completed the study without any dropout. Baseline characteristics of patients are shown in Table 1. The mean and standard deviation of premorbid IQ assessed by the Japanese Adult Reading Test (25) was 99.6 (12.0). The mean standard deviation of the global assessment of functioning (26) was 38.6 (6.9). Antipsychotics taken by participants were as follows: risperidone (eight patients), paliperidone, quetiapine, aripiprazole (seven for each), olanzapine (six), haloperidol (three), chlorpromazine, levomepromazine, zotepine (two for each), perospirone, blonanserin, sulpiride (one for each). No medication was modified during the study period. No severe side effect was observed throughout the trial. All participants tolerated the treatment well.

**Table 1:** Clinical characteristics of patients (*n* = *28*).

Variables	Mean ± SD or *n*
Inpatient/outpatient	*22*/*6*
Male/female	*16*/*12*
Age, year	40.9 ± 9.8
Age at onset, year	23.6 ± 6.7
Duration of present illness, year	17.3 ± 9.9
Chlorpromazine equivalent dose of antipsychotics, mg/day	889.0 ± 587.1
Duration of education, year	13.8 ± 1.7
Premorbid IQ	99.6 ± 12.0
Global assessment of functioning	38.6 ± 6.9

### Intervention

We used a Soterix Medical 1 × 1 Transcranial Direct Current Low-Intensity Stimulator Model 1,300 A. For each session, the tDCS montage comprised placement of the anode over the left DLPFC and the cathode over the right supraorbital area (corresponding to F3 and FP2, according to the International 10–20 electroencephalography system), as previously investigated (27). Rubber electrodes were inserted in 35-cm^2^ saline-soaked sponges, and were fixed with headband. We applied direct current of 2 mA for 20 min per session. Subjects underwent 10 tDCS sessions in 5 consecutive days, twice per day. On each day, tDCS intervention was performed approximately at 10 a.m. and 2 p.m.

Trained psychiatrists administered tDCS intervention. In order to maximize adherence, we provided all included patients and their study partners with costs of transportation, and reminded and rescheduled all of the patients’ visits if necessary.

Criteria for discontinuing interventions were as follows:

(1) In case patients withdraw informed consent to participate.
(2) In case severe adverse effects are observed.
(3) In case patients fail to undergo three consecutive sessions of tDCS.

Adjusting drugs were considered as a protocol deviation during the trial.

### Outcome Measures

Subjects were assessed at baseline and 1 month after the last stimulation. Each evaluation was performed by experienced psychologists (Kazuki Sueyoshi and Crystal Lin). They were trained at a workshop.

#### Cognition

Cognitive function was assessed by the BACS. It is used to evaluate cognitive domains that are typically impaired in patients with schizophrenia, including verbal memory (Verbal Memory Task), verbal working memory (Digit Sequencing Task), motor/speed (Token Motor Task), verbal fluency (Verbal Fluency Task), attention/information processing (Symbol Coding Task), and executive function (Tower of London Task) (28). The higher scores represent better cognition. To provide a standard metric for combining test scores into domains and comparing performance over time, BACS scores were converted to *z*-scores (continuous variables) which show performances relative to those of healthy people (5). Alternative forms were used for the Verbal Memory Task and Tower of London Task at baseline and follow-up assessments.

#### Functional Capacity (Daily Living Skills)

Functional capacity was assessed by the UPSA-B (29). It is one of the measures most frequently used to evaluate daily living skills linked to cognitive function in schizophrenia (9, 29). Patients performed worse on the UPSA-B than do healthy individuals, a finding pertinent to some of the non-Western countries including Japan (9, 29). The UPSA-B consists of finance and communication subscales, which are continuous variables. Subscale scores of the two domains of the UPSA-B were converted into standard scores ranging from 0 to 50, so that the maximum of the total score was 100 (30). The higher scores represent better functional capacity. The validity of its Japanese version was confirmed (29).

#### Psychotic Symptoms

Psychotic symptoms were evaluated by the positive and negative syndrome scale (PANSS), commonly used for the assessment of psychotic symptoms of schizophrenia (31). The PANSS is a structured interview, consisting of positive, negative, and general psychopathology subscales (with scores ranging from 7 to 49, from 7 to 49, and from 16 to 112, respectively), whose scores are regarded as continuous variables. The higher scores represent more severe psychotic symptoms.

#### Depression

The Calgary depression scale for schizophrenia (CDSS), recommended as a brief and reliable tool for the assessment of severity of depression in schizophrenia (32), was used to assess depressive symptoms. The CDSS is a structured interview, consisting of items for depression, hopelessness, self-depreciation, guilty ideas of reference, pathological guilt, morning depression, early wakening, suicide, and observed depression. The score of each item ranges from 0 to 3 (i.e., discrete variable) (32). The higher scores represent more severe depressive state.

This study was approved by Ethical Committee of National Center of Neurology and Psychiatry, Tokyo, Japan. This was a single-arm, open-label study in which outcome measures were carried out before and after tDCS intervention (UMIN000015953). The patients first underwent a baseline assessment of the BACS, UPSA-B, PANSS, and CDSS; then they participated in the stimulation protocol consisting in twice-daily (10 a.m. and 2 p.m.) anodal tDCS over the left DLPFC and cathodal tDCS on the right superorbital area for 5 days, and were assessed again 1 month after the last stimulation. The study schedule is summarized in Table 2.

**Table 2:** Study schedule.

	Study period		
	Enrollment	Intervention	Follow-up

Time point	Week 1	Week 2 (5 consecutive days)	Week 7
**Enrollment**			
Eligibility screen	X		
Informed consent	X		
Sociodemographic characteristics	X		
**Intervention**			
tDCS (twice/day)		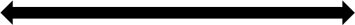	
**Assessments**			
BACS	X		X
UPSA-B	X		X
PANSS	X		X
CDSS	X		X
Adverse events	X	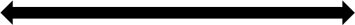	X

*BACS, the brief assessment for cognition in schizophrenia; UPSA-B, the UCSD performance-based skills assessment-brief; PANSS, positive and negative syndrome scale; CDSS, Calgary depression scale for schizophrenia*.

### Statistical Analysis

Correlations between baseline values and their changes from baseline of BACS, UPSA-B, PANSS, and CDSS scores, were evaluated. Correlations were also examined for chlorpromazine equivalent dose of antipsychotics vs. changes from baseline of BACS, UPSA-B, PANSS, and CDSS scores, as well as change from baseline of UPSA-B scores vs. changes from baseline of BACS, PANSS, and CDSS scores.

Statistical analysis was conducted using STATA 14, created by StataCorp in TX, USA. We performed a per protocol approach for subjects who were followed-up until the end of study point. For continuous variables in the BACS, UPSA-B, PANSS, we used Student’s *t*-test. For a discrete variable in the CDSS, we performed Wilcoxon signed-rank test. Pearson’s product moment correlation coefficient was used for the relationship between clinical variables.

### Monitoring

A systematic review revealed that the most common adverse events were itching, tingling, headache, burning sensation, and discomfort (33). A trained psychiatrist evaluated the safety with a semistructured checklist of these symptoms after each intervention. An independent safety monitoring committee ran an interim analysis for safety every week.

## Results

Table 3 shows outcome measures at baseline and 1 month after the last administration of tDCS.

**Table 3:** Outcome measures at baseline and 1 month after the treatment.

	Baseline, mean ± SD	Follow-up, mean ± SD	*t*-Value (degree of freedom) or *z*-value	*p*-Value	Effect size
**BACS (*z*-score)**					
Composite score	−1.86 ± 0.92	−1.40 ± 0.93	*t* = 4.23 (27)	**<0.001**	***d* = 0.49**
Verbal memory	−1.67 ± 1.06	−1.06 ± 1.14	*t* = 4.53 (27)	**<0.001**	***d* = 0.55**
Digit sequencing	−1.16 ± 1.38	−0.95 ± 1.37	*t* = 1.52 (27)	0.14	*d* = 0.15
Token motor	−3.27 ± 1.25	−2.73 ± 1.23	*t* = 2.47 (27)	**0.020**	***d* = 0.44**
Verbal fluency	−1.19 ± 1.05	−0.84 ± 0.89	*t* = 2.10 (27)	**0.046**	***d* = 0.36**
Symbol coding	−2.25 ± 1.22	−2.21 ± 1.44	*t* = 0.25 (27)	0.80	*d* = 0.03
Tower of London	−1.76 ± 2.03	−1.12 ± 2.16	*t* = 1.88 (27)	0.071	*d* = 0.31
**UPSA-B**					
Total	68.4 ± 14.8	79.0 ± 15.5	*t* = 5.89 (27)	**<0.001**	***d* = 0.70**
Finance	41.4 ± 8.1	45.8 ± 6.2	*t* = 3.35 (27)	**0.002**	***d* = 0.61**
Communication	27.1 ± 9.6	33.2 ± 11.1	*t* = 3.57 (27)	**0.001**	***d* = 0.59**
**PANSS**					
Positive syndrome	15.7 ± 5.7	13.1 ± 4.8	*t* = 2.31 (27)	**0.029**	***d* = 0.48**
Negative syndrome	14.9 ± 8.0	13.6 ± 6.7	*t* = 1.24 (27)	0.23	*d* = 0.17
General psychopathology	32 ± 8.1	28.3 ± 7.1	*t* = 2.35 (27)	**0.027**	***d* = 0.50**
**CDSS**					
Total	8.00 ± 4.97	5.36 ± 3.89	*z* = 2.83	**0.005**	***r* = 0.38**
Depression	0.79 ± 0.79	0.89 ± 0.92	*z* = 0.79	0.43	*r* = −0.11
Hopelessness	0.86 ± 0.93	0.57 ± 0.74	*z* = 1.58	0.11	*r* = 0.21
Self-depreciation	1.21 ± 1.17	0.71 ± 0.85	*z* = 2.46	**0.014**	***r* = 0.33**
Guilty ideas of reference	0.75 ± 1.04	0.50 ± 0.64	*z* = 0.39	0.39	*r* = 0.11
Pathological guilt	0.82 ± 0.90	0.57 ± 0.88	*z* = 1.58	0.11	*r* = 0.21
Morning depression	0.82 ± 0.72	0.61 ± 0.63	*z* = 2.12	**0.034**	***r* = 0.28**
Early wakening	1.68 ± 1.25	1.00 ± 0.86	*z* = 3.11	**0.002**	***r* = 0.42**
Suicide	0.61 ± 0.83	0.25 ± 0.52	*z* = 1.99	**0.046**	***r* = 0.27**
Observed depression	0.46 ± 0.51	0.25 ± 0.44	*z* = 1.90	0.058	*r* = 0.25

*BACS, The Brief Assessment for Cognition in Schizophrenia; UPSA-B, The UCSD Performance-based Skills Assessment-Brief; PANSS, Positive and Negative Syndrome Scale; CDSS, Calgary Depression Scale for Schizophrenia*.

### Cognition

Significant improvement was found on BACS composite scores (*t* = 4.23, *p* < 0.001), as well as on verbal memory (*t* = 4.53, *p* < 0.001), motor/speed (*t* = 2.47, *p* = 0.020), and verbal fluency (*t* = 2.10, *p* = 0.046) subtests. Improvement of verbal memory was associated with a largest effect size (*d* = 0.55), while small to medium effect sizes were noted for motor/speed (*d* = 0.44), verbal fluency (*d* = 0.36), and composite scores (*d* = 0.49). No significant improvement was found on working memory, attention/information processing, and executive function.

### Functional Capacity (Daily Living Skills)

Significant improvement was noted on UPSA-B finance (*t* = 3.35, *p* = 0.002) and communication (*t* = 3.57, *p* = 0.001) subscale scores, as well as on total scores (*t* = 5.89, *p* < 0.001), with medium to large effect sizes (*d* = 0.61, *d* = 0.59, and *d* = 0.70, respectively).

### Psychotic Symptoms

Significant improvement was found on PANSS positive (*t* = 2.31, *p* = 0.029) and general psychopathology (*t* = 2.35, *p* = 0.027) subscale scores, with medium effect sizes (*d* = 0.48 and *d* = 0.58, respectively). On the other hand, no significant improvement was found for negative syndrome subscale scores.

### Depression

Significant improvement was demonstrated on self-depreciation (*z* = 2.46, *p* = 0.014), morning depression (*z* = 2.12, *p* = 0.034), early wakening (*z* = 3.11, *p* = 0.002), and suicide (*z* = 1.99, *p* = 0.046) item scores, as well as total scores (*z* = 2.83, *p* = 0.005) of the CDSS, with small to medium effect sizes (*r* = 0.33, *r* = 0.28, *r* = 0.42, *r* = 0.27, and *r* = 0.38, respectively). On the other hand, depression, hopelessness, guilty ideas of reference, pathological guilt, and observed depression items were not significantly changed.

### Correlation

**Figure 1:**
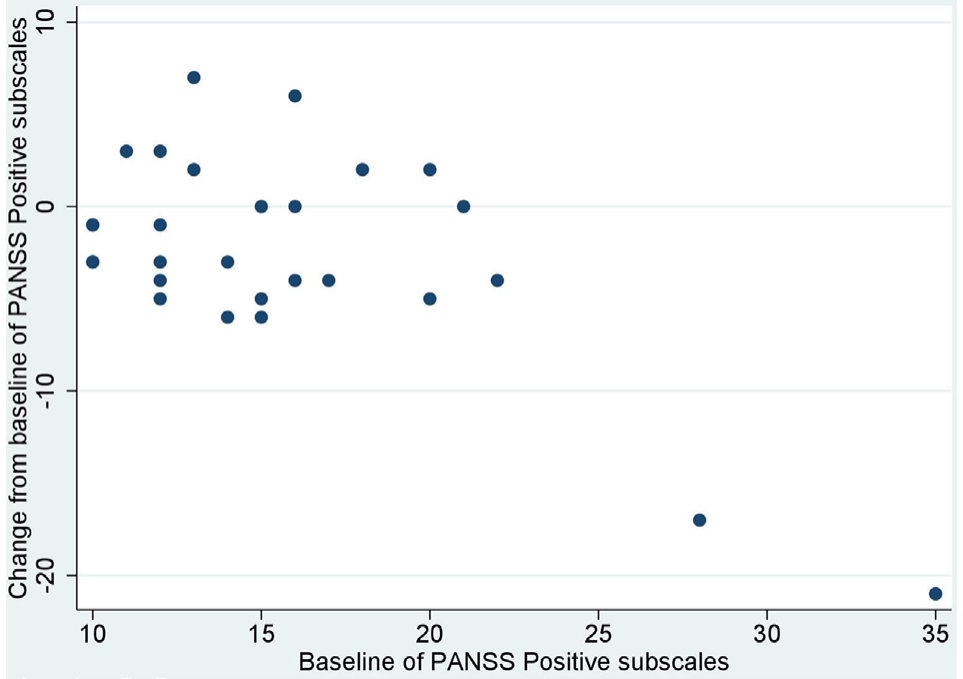
Correlation between the baseline and change from baseline of positive and negative syndrome scale (PANSS) positive subscales (*r* = −0.65, *p* < 0.001).

**Figure 2:**
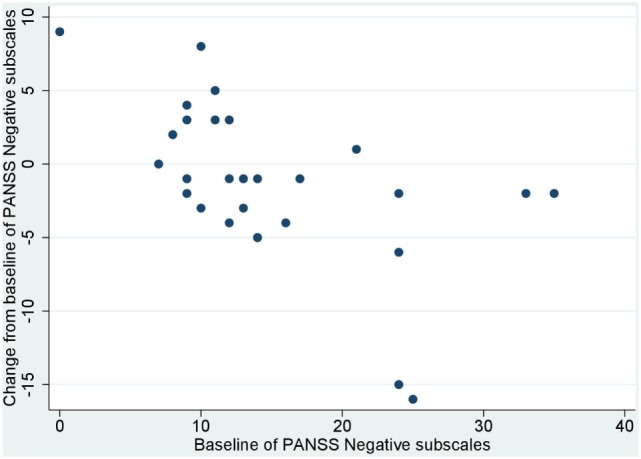
Correlation between the baseline and change from baseline of positive and negative syndrome scale (PANSS) negative subscales (*r* = −0.56, *p* < 0.002).

**Figure 3:**
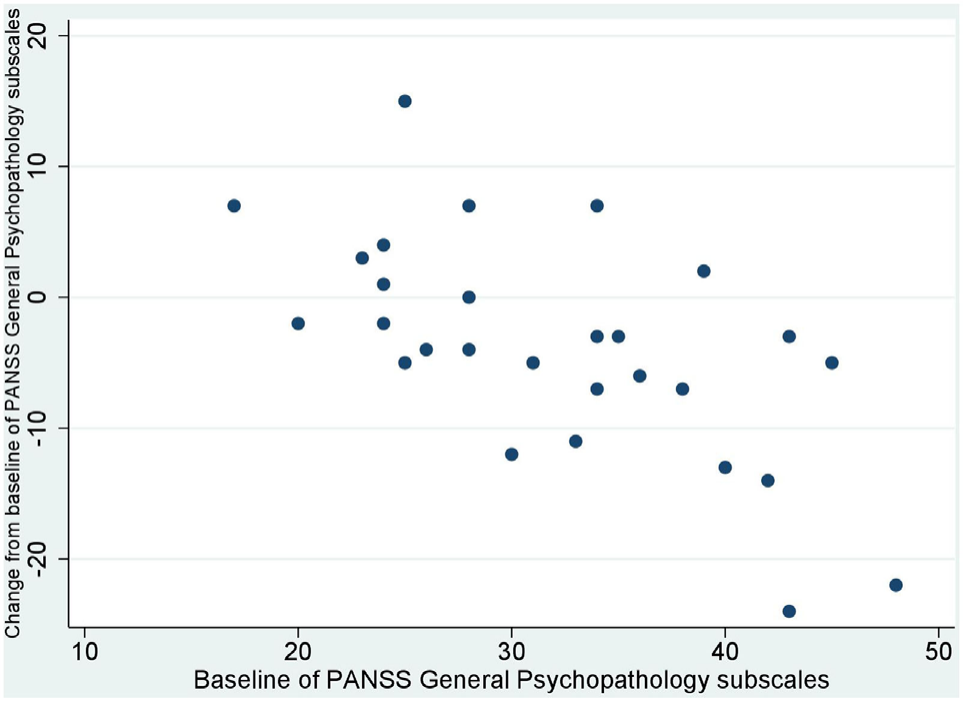
Correlation between the baseline and change from baseline of positive and negative syndrome scale (PANSS) general psychopathology subscales (*r* = −0.64, *p* < 0.001).

**Figure 4:**
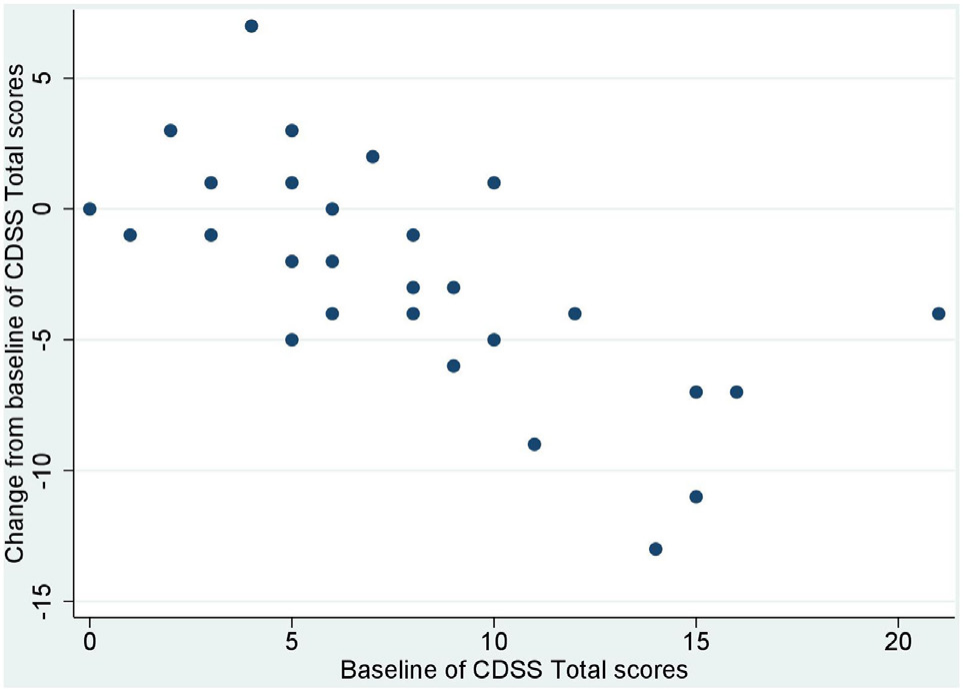
Correlation between the baseline and change from baseline of Calgary depression scale for schizophrenia (CDSS) total scores (*r* = −0.66, *p* < 0.001).

No significant correlation was noted between baseline values and their changes from baseline of BACS and UPSA-B scores. In contrast, significant negative correlations were demonstrated between baseline values vs. their changes from baseline of PANSS positive subscales (*r* = −0.65, *p* < 0.001, Figure 1), negative subscales (*r* = −0.56, *p* < 0.002, Figure 2), general psychopathology subscales (*r* = −0.64, *p* < 0.001, Figure 3), and CDSS total scores (*r* = −0.66, *p* < 0.001, Figure 4). No significant correlation was found between chlorpromazine equivalent dose of antipsychotics vs. changes from baseline of BACS, UPSA-B, PANSS, and CDSS scores. The same applied to correlations between change from baseline of UPSA-B scores vs. changes from baseline of BACS, PANSS, and CDSS scores.

## Discussion

To our knowledge, this study was the first to suggest the ability of tDCS to improve daily living skills linked to cognition (functional capacity, measured by UPSA-B), as well as depressive symptoms (measured by the CDSS) in patients with schizophrenia. Also, this study was the first to indicate the improvement of functional capacity after 5-day administration of tDCS, which was not correlated with the change of cognition, psychosis, and depression. At the same time, tDCS was found to enhance cognition in these subjects.

The results obtained in this trial are consistent with those from other studies indicating that tDCS may be effective to improve cognition in healthy controls (34–36) and patients with schizophrenia (18, 22, 23, 37). In a previous study, five sessions of tDCS in consecutive days, with the anode over F3 and the cathode over FP2, enhanced working memory and attention/vigilance to a greater extent than did sham-treatment in patients with schizophrenia (24). In our study, significant effects of tDCS were noted in the domains of verbal memory (with different versions of word lists at baseline and follow-up), with a small to medium effect size, which suggests that tDCS moderately promotes cognitive function in schizophrenia. On the other hand, no significant improvement was demonstrated in working memory. This discrepancy may be due to the difference in the study design, treatment regimen (5 session of tDCS in consecutive days in the previous study, while 10 sessions of tDCS in 5 consecutive days in our study), and/or sample size. It is also possible that an effect on working memory might have vanished in 1-month follow-up period. In this sense, the inclusion of data on the cognitive outcomes immediately after the last stimulation could have provided more detailed information. While a previous study evaluated working memory immediately after the last tDCS (24, 38), the measurement of the BACS and UPSA-B at this time-point were not included in our protocol. This was because evaluating the BACS and UPSA-B just 1 week after baseline assessments with the same tests could produce learning effects.

The mechanisms by which tDCS affects cognitive function in schizophrenia may be explained in several ways. Functional connectivity of the frontoparietal control network and interhemispheric connectivity are decreased in schizophrenia patients, likely to be related to impairment of higher order cognitive task-related activities and disruption of the default mode network (10–12). Several studies demonstrated that tDCS alters functional connectivity in, for example, the default mode network and the frontoparietal control network, in healthy subjects (39, 40). Also, a case study reported that tDCS changed functional brain connectivity in the anterior part of the default mode network (41). Furthermore, Kim et al. reported neurophysiological evidence that tDCS modulates sensory gating in schizophrenia (42). Neurochemically, the after-effects of anodal tDCS are considered to depend on modulation of both GABAergic and glutamatergic synaptic transmissions (17). Further investigations of biological measures are warranted to elucidate the mechanisms by which tDCS exerts pro-cognitive effects.

tDCS was also found to improve daily living skills linked to cognition (functional capacity), measured by the UPSA-B with medium to large effect sizes, in schizophrenia. To our knowledge, there has been no attempt to elucidate the effect of tDCS on this level of functional outcomes in psychiatric conditions. In view of the association between performance on the UPSA-B and real-world functional outcomes (43), the result reported here suggests the ability of tDCS to enhance social outcome in schizophrenia.

Data from this study also suggest the ability of tDCS to ameliorate depressive mood, evaluated objectively by the CDSS (32), in patients with schizophrenia. Results from a meta-analysis indicate that tDCS is effective in treating patients with major depressive disorder, with an effect size comparable with those reported for repetitive transcranial magnetic stimulation and antidepressant drugs (44). Some domains in depressive symptoms were improved with small to medium effect sizes, similar to the case in patients with major depressive disorder (44). The antidepressant effect of tDCS may be related to hypoactivity of the left DLPFC, which is likely to be restored by anodal tDCS (45). The results presented in the current study are consistent with this hypothesis, and may provide a strategy to ameliorate treatment-resistant depressive symptoms in patients with schizophrenia.

tDCS was found to improve positive symptoms and general psychopathology with medium effect sizes, which is advantageous for patients suffering from psychotic symptoms. So far, two studies have attempted to see the effect of tDCS on psychopathology, as measured by the PANSS. Brunelin et al. did not find a significant effect on either positive or negative symptoms. These authors placed the anodal electrode over a point midway between F3 and FP1 and the cathodal electrode over a point midway between T3 and P3 (46). On the other hand, Smith et al. observed significant improvement only in negative symptoms. These investigators placed the anode over F3 and the cathode over FP2 (24). Taken together, further studies to seek optimal methods of tDCS to ameliorate psychotic symptoms are needed.

Changes of cognition and daily living skills were not correlated with their baseline scores. In contrast, the improvements of positive symptoms, negative symptoms, general psychopathology, and depression were correlated with their baseline scores. These observations suggest the lack of ceiling effects of tDCS on cognition and daily living skills. Also, the lack of significant correlation between chlorpromazine equivalent dose of antipsychotics vs. the improvement of cognition, functional outcome, psychotic symptoms, and depression suggests that tDCS may improve these outcome measures regardless of the dose of antipsychotics. Furthermore, the lack of significant correlation between the improvement of functional capacity vs. cognition, positive symptoms, and depression, indicates that the observed change of functional capacity was independent of these clinical variables. However, the possibility of unobserved confounders cannot be ruled out completely with the current study design.

The limitations of this study should be noted here. The lack of blinding might have produced practice (repeated-measure) effect in some measures used. To circumvent this issue, alternate forms were used for verbal memory (word list learning task) and executive function (Tower of London task) in the BACS at the follow-up assessment. Therefore, the pro-cognitive effect of tDCS on verbal memory may not be attributable to repeated-measure effect. In addition, a small sample of this study may raise caution in concluding that these results represent effects in the population. Also, the lack of randomization, controlled group, and blinding might have produced placebo effects. Inclusion of a sham-controlled group could have provided a definitive conclusion. Accordingly, we are initiating a randomized sham-controlled trial with a larger sample (Narita, et al. submitted; UMIN000028224).

In conclusion, the results of the present study suggest the efficacy of tDCS on cognition, daily living skills, and depression. These results may add to the concept that tDCS provides a strategy to enhance functional outcomes in patients with schizophrenia.

## Ethics Statement

This study was carried out in accordance with the recommendations of Ethical Guideline for Clinical Researches, Ministry of Health, Labor and Welfare with written informed consent from all subjects. All subjects gave written informed consent in accordance with the Declaration of Helsinki. The protocol was approved by the Ethical Committee of National Center of Neurology and Psychiatry.

## Author Contributions

ZN managed the literature searches, undertook the statistical analysis, and wrote the first draft of the manuscript. TI, TS, and ZN administered tDCS. TS designed the study and wrote the protocol. CL and KS conducted clinical assessments. All authors made substantial contribution, drafted the manuscript, and approved the final manuscript.

## Conflict of Interest Statement

The authors declare that the research was conducted in the absence of any commercial or financial relationships that could be construed as a potential conflict of interest.

## References

[1] Kremen WS Vinogradov S Poole JH Schaefer CA Deicken RF Factor-Litvak P Cognitive decline in schizophrenia from childhood to midlife: a 33-year longitudinal birth cohort study. Schizophr Res (2010) 118(1–3):1–5. 10.1016/j.schres.2010.01.009 20153140 PMC3184642

[2] Kurtz MM. Neurocognitive impairment across the lifespan in schizophrenia: an update. Schizophr Res (2005) 74(1):15–26. 10.1016/j.schres.2004.07.005 15694750

[3] Micallef J Fakra E Blin O [Use of antidepressant drugs in schizophrenic patients with depression]. Encephale (2006) 32(2 Pt 1):263–9. 10.1016/S0013-7006(06)76153-X 16910628

[4] Saykin AJ Shtasel DL Gur RE Kester DB Mozley LH Stafiniak P Neuropsychological deficits in neuroleptic naive patients with first-episode schizophrenia. Arch Gen Psychiatry (1994) 51(2):124–31. 10.1001/archpsyc.1994.03950020048005 7905258

[5] Saykin AJ Gur RC Gur RE Mozley PD Mozley LH Resnick SM Neuropsychological function in schizophrenia. Selective impairment in memory and learning. Arch Gen Psychiatry (1991) 48(7):618–24. 10.1001/archpsyc.1991.01810310036007 2069492

[6] Green MF Nuechterlein KH Kern RS Baade LE Fenton WS Gold JM Functional co-primary measures for clinical trials in schizophrenia: results from the MATRICS psychometric and standardization study. Am J Psychiatry (2008) 165(2):221–8. 10.1176/appi.ajp.2007.07010089 18172017

[7] Harvey PD Velligan DI International assessment of functional skills in people with schizophrenia. Innov Clin Neurosci (2011) 8(1):15–8. PMC3036557 21311703

[8] Harvey PD Raykov T Twamley EW Vella L Heaton RK Patterson TL. Validating the measurement of real-world functional outcomes: phase I results of the VALERO study. Am J Psychiatry (2011) 168(11):1195–201. 10.1176/appi.ajp.2011.10121723 21572166 PMC3670945

[9] Sumiyoshi T Nishida K Niimura H Toyomaki A Morimoto T Tani M Cognitive insight and functional outcome in schizophrenia; a multi-center collaborative study with the specific level of functioning scale – Japanese version. Schizophr Res Cogn (2016) 6:9–14. 10.1016/j.scog.2016.08.001 28740819 PMC5514305

[10] Baker JT Holmes AJ Masters GA Yeo BTT Krienen F Buckner RL Disruption of cortical association networks in schizophrenia and psychotic bipolar disorder. JAMA Psychiatry (2014) 71(2):109–18. 10.1001/jamapsychiatry.2013.3469 24306091 PMC4435541

[11] Guo W Xiao C Liu G Wooderson SC Zhang Z Zhang J Decreased resting-state interhemispheric coordination in first-episode, drug-naive paranoid schizophrenia. Prog Neuropsychopharmacol Biol Psychiatry (2014) 48:14–9. 10.1016/j.pnpbp.2013.09.012 24075897

[12] Hoptman MJ Zuo X-N D’Angelo D Mauro CJ Butler PD Milham MP Decreased interhemispheric coordination in schizophrenia: a resting state fMRI study. Schizophr Res (2012) 141(1):1–7. 10.1016/j.schres.2012.07.027 22910401 PMC3446206

[13] Sheffield JM Repovs G Harms MP Carter CS Gold JM MacDonald AW Fronto-parietal and cingulo-opercular network integrity and cognition in health and schizophrenia. Neuropsychologia (2015) 73:82–93. 10.1016/j.neuropsychologia.2015.05.006 25979608 PMC4505838

[14] Sheffield JM Barch DM Cognition and resting-state functional connectivity in schizophrenia. Neurosci Biobehav Rev (2016) 61:108–20. 10.1016/j.neubiorev.2015.12.007 26698018 PMC4731300

[15] Yokoi Y Narita Z Sumiyoshi T Transcranial direct current stimulation in depression and psychosis: a systematic review. Clin EEG Neurosci (2017) (in press). 10.1177/1550059417732247 28929795

[16] Yokoi Y Sumiyoshi T Application of transcranial direct current stimulation to psychiatric disorders: trends and perspectives. Neuropsychiatr Electrophysiol (2015) 1:10 10.1186/s40810-015-0012-x

[17] Stagg CJ Nitsche MA. Physiological basis of transcranial direct current stimulation. Neuroscientist (2011) 17(1):37–53. 10.1177/1073858410386614 21343407

[18] Schretlen DJ van Steenburgh JJ Varvaris M Vannorsdall TD Andrejczuk MA Gordon B. Can transcranial direct current stimulation improve cognitive functioning in adults with schizophrenia? Clin Schizophr Relat Psychoses (2014) 3:1–27. 10.3371/CSRP.SCST.103114 25367166

[19] Sumiyoshi T Higuchi Y. Facilitative effect of serotonin(1A) receptor agonists on cognition in patients with schizophrenia. Curr Med Chem (2013) 20(3):357–62. 10.2174/092986713804870846 23157627

[20] Stagg CJ Lin RL Mezue M Segerdahl A Kong Y Xie J Widespread modulation of cerebral perfusion induced during and after transcranial direct current stimulation applied to the left dorsolateral prefrontal cortex. J Neurosci (2013) 33(28):11425–31. 10.1523/JNEUROSCI.3887-12.2013 23843514 PMC3724554

[21] Lorenz J Minoshima S Casey KL. Keeping pain out of mind: the role of the dorsolateral prefrontal cortex in pain modulation. Brain (2003) 126(Pt 5):1079–91. 10.1093/brain/awg102 12690048

[22] Vercammen A Rushby JA Loo C Short B Weickert CS Weickert TW Transcranial direct current stimulation influences probabilistic association learning in schizophrenia. Schizophr Res (2011) 131(1–3):198–205. 10.1016/j.schres.2011.06.021 21745726

[23] Hoy KE Bailey NW Arnold SL Fitzgerald PB. The effect of transcranial direct current stimulation on gamma activity and working memory in schizophrenia. Psychiatry Res (2015) 228(2):191–6. 10.1016/j.psychres.2015.04.032 25997999

[24] Smith RC Boules S Mattiuz S Youssef M Tobe RH Sershen H Effects of transcranial direct current stimulation (tDCS) on cognition, symptoms, and smoking in schizophrenia: a randomized controlled study. Schizophr Res (2015) 168(1–2):260–6. 10.1016/j.schres.2015.06.011 26190299

[25] Fukue T Fukue M Ishizuka Y Relationship between Japanese adult reading test (JART) and cognitive dysfunction. Jpn J Gen Hosp Psychiatry (2013) 25(1):55–62.

[26] Hall RC. Global assessment of functioning. A modified scale. Psychosomatics (1995) 36(3):267–75. 10.1016/S0033-3182(95)71666-8 7638314

[27] Boggio PS Rigonatti SP Ribeiro RB Myczkowski ML Nitsche MA Pascual-Leone A A randomized, double-blind clinical trial on the efficacy of cortical direct current stimulation for the treatment of major depression. Int J Neuropsychopharmacol (2008) 11(2):249–54. 10.1017/S1461145707007833 17559710 PMC3372849

[28] Segarra N Bernardo M Gutierrez F Justicia A Fernadez-Egea E Allas M Spanish validation of the brief assessment in cognition in schizophrenia (BACS) in patients with schizophrenia and healthy controls. Eur Psychiatry (2011) 26(2):69–73. 10.1016/j.eurpsy.2009.11.001 20435446

[29] Sumiyoshi C Takaki M Okahisa Y Patterson TL Harvey PD Sumiyoshi T Utility of the UCSD performance-based skills assessment-brief Japanese version: discriminative ability and relation to neurocognition. Schizophr Res Cogn (2014) 1(3):137–43. 10.1016/j.scog.2014.08.002 PMC5779073 29379746

[30] Mausbach BT Harvey PD Goldman SR Jeste DV Patterson TL. Development of a brief scale of everyday functioning in persons with serious mental illness. Schizophr Bull (2007) 33(6):1364–72. 10.1093/schbul/sbm014 17341468 PMC2779885

[31] Kay SR Opler LA Lindenmayer JP. Reliability and validity of the positive and negative syndrome scale for schizophrenics. Psychiatry Res (1988) 23(1):99–110. 10.1016/0165-1781(88)90038-8 3363019

[32] Bernard D Lançon C Auquier P Reine G Addington D. Calgary depression scale for schizophrenia: a study of the validity of a French-language version in a population of schizophrenic patients. Acta Psychiatr Scand (1998) 97(1):36–41. 10.1111/j.1600-0447.1998.tb09960.x 9504701

[33] Brunoni AR Amadera J Berbel B Volz MS Rizzerio BG Fregni F. A systematic review on reporting and assessment of adverse effects associated with transcranial direct current stimulation. Int J Neuropsychopharmacol (2011) 14(8):1133–45. 10.1017/S1461145710001690 21320389

[34] Brasil-Neto JP. Learning, memory, and transcranial direct current stimulation. Front Psychiatry (2012) 3:80. 10.3389/fpsyt.2012.00080 22969734 PMC3432476

[35] Jeon SY Han SJ. Improvement of the working memory and naming by transcranial direct current stimulation. Ann Rehabil Med (2012) 36(5):585–95. 10.5535/arm.2012.36.5.585 23185722 PMC3503933

[36] Nelson JT McKinley RA Golob EJ Warm JS Parasuraman R. Enhancing vigilance in operators with prefrontal cortex transcranial direct current stimulation (tDCS). Neuroimage (2014) 85(Pt 3):909–17. 10.1016/j.neuroimage.2012.11.061 23235272

[37] Tarur Padinjareveettil AM Rogers J Loo C Martin D Transcranial direct current stimulation to enhance cognitive remediation in schizophrenia. Brain Stimulat (2015) 8(2):307–9. 10.1016/j.brs.2014.11.012 25547192

[38] Fröhlich F Burrello TN Mellin JM Cordle AL Lustenberger CM Gilmore JH Exploratory study of once-daily transcranial direct current stimulation (tDCS) as a treatment for auditory hallucinations in schizophrenia. Eur Psychiatry (2016) 33:54–60. 10.1016/j.eurpsy.2015.11.005 26866874

[39] Keeser D Meindl T Bor J Palm U Pogarell O Mulert C Prefrontal transcranial direct current stimulation changes connectivity of resting-state networks during fMRI. J Neurosci (2011) 31(43):15284–93. 10.1523/JNEUROSCI.0542-11.2011 22031874 PMC6703525

[40] Peña-Gómez C Sala-Lonch R Junqué C Clemente IC Vidal D Bargalló N Modulation of large-scale brain networks by transcranial direct current stimulation evidenced by resting-state functional MRI. Brain Stimulat (2012) 5(3):252–63. 10.1016/j.brs.2011.08.006 21962981 PMC3589751

[41] Palm U Keeser D Blautzik J Pogarell O Ertl-Wagner B Kupka MJ Prefrontal transcranial direct current stimulation (tDCS) changes negative symptoms and functional connectivity MRI (fcMRI) in a single case of treatment-resistant schizophrenia. Schizophr Res (2013) 150(2–3):583–5. 10.1016/j.schres.2013.08.043 24060570

[42] Kim M Yoon YB Lee TH Lee TY Kwon JS The effect of tDCS on auditory hallucination and P50 sensory gating in patients with schizophrenia: a pilot study. Schizophr Res (2017). 10.1016/j.schres.2017.04.023 28416094

[43] Sumiyoshi C Harvey PD Takaki M Okahisa Y Sato T Sora I Factors predicting work outcome in Japanese patients with schizophrenia: role of multiple functioning levels. Schizophr Res Cogn (2015) 2(3):105–12. 10.1016/j.scog.2015.07.003 PMC5779305 29379760

[44] Brunoni AR Moffa AH Fregni F Palm U Padberg F Blumberger DM Transcranial direct current stimulation for acute major depressive episodes: meta-analysis of individual patient data. Br J Psychiatry (2016) 208(6):522–31. 10.1192/bjp.bp.115.164715 27056623 PMC4887722

[45] Brunoni AR Teng CT Correa C Imamura M Brasil-Neto JP Boechat R Neuromodulation approaches for the treatment of major depression: challenges and recommendations from a working group meeting. Arq Neuropsiquiatr (2010) 68(3):433–51. 10.1590/S0004-282X2010000300021 20602051

[46] Brunelin J Mondino M Gassab L Haesebaert F Gaha L Suaud-Chagny M-F Examining transcranial direct-current stimulation (tDCS) as a treatment for hallucinations in schizophrenia. Am J Psychiatry (2012) 169(7):719–24. 10.1176/appi.ajp.2012.11071091 22581236

